# P01.29. Pre-polarization conductance at Jing-Well acupoints on the hand is correlated with blood flow measured by laser doppler flowmetry

**DOI:** 10.1186/1472-6882-12-S1-P29

**Published:** 2012-06-12

**Authors:** S Lin, G Orenstein, A Froloff, N Nguyen, D Unsworth, A Samadi, J Gartner

**Affiliations:** 1 University of California, Irvine, Irvine, USA

## Purpose

“Blood is the mother of Qi” is a fundamental concept of Traditional Chinese Medicine. Since Qi is not a scientific term, we explored this relationship by measuring electrical flow as a surrogate marker when blood flow is changing under different conditions.

## Methods

This study involved a dozen healthy subjects (age 20's to 60’s). Blood flow/perfusion was measured with a Moor DRT4 laser Doppler flowmetry instrument. Electrical flow/pre-polarization conductance was measured with Motoyama’s "single square voltage pulse" method using a continuous recording instrument kindly provided by California Institute for Human Science.

## Results

We found that during Chen style Tai Chi “silk-reeling” exercises, the cyclic down/up hand movement (2X/minute) caused cycles of increase/decrease in blood flow measured at PC8 acupoint and electrical conductance at 7 jing-well acupoints on the hand. Similar results were obtained when subjects performed dance movements with similar down/up cycles of the hand. We also observed that cyclic increase/decrease in blood flow induced by subjects with arm fully stretched upwards being rocked by an assistant down/up on an inversion exercise table was accompanied by cyclic increase/decrease in conductance. During the above active or passive hand movement cycles, when blood flow to the hand was cut off by a pressure cuff on the arm, laser Doppler and conductance values both dropped to baseline, confirming lack of motion artifacts. Furthermore the cyclic changes in “flux” in all experiments coincided with “speed” values from laser Doppler flowmetry and with pre-polarization but not post-polarization conductance (galvanic skin response) measured at the acupoints.

## Conclusion

This study shows that the increase/decrease in blood flow caused by gravitational pull on the blood in the hand as its level decreases/increases relative to the heart is accompanied by corresponding changes in electrical flow at acupoints, consistent with the belief in Traditional Chinese Medicine of a close relationship between blood and Qi.

